# IRF4 Has a Unique Role in Early B Cell Development and Acts Prior to CD21 Expression to Control Marginal Zone B Cell Numbers

**DOI:** 10.3389/fimmu.2021.779085

**Published:** 2021-11-22

**Authors:** Kristina Ottens, Anne B. Satterthwaite

**Affiliations:** ^1^ Department of Internal Medicine, University of Texas (UT) Southwestern Medical Center, Dallas, TX, United States; ^2^ Department of Immunology, University of Texas (UT) Southwestern Medical Center, Dallas, TX, United States

**Keywords:** B cell development, IRF4, IRF8, IL-7, marginal zone B cell

## Abstract

Strict control of B lymphocyte development is required for the ability to mount humoral immune responses to diverse foreign antigens while remaining self-tolerant. In the bone marrow, B lineage cells transit through several developmental stages in which they assemble a functional B cell receptor in a stepwise manner. The immunoglobulin heavy chain gene is rearranged at the pro-B stage. At the large pre-B stage, cells with a functional heavy chain expand in response to signals from IL-7 and the pre-BCR. Cells then cease proliferation at the small pre-B stage and rearrange the immunoglobulin light chain gene. The fully formed BCR is subsequently expressed on the surface of immature B cells and autoreactive cells are culled by central tolerance mechanisms. Once in the periphery, transitional B cells develop into mature B cell subsets such as marginal zone and follicular B cells. These developmental processes are controlled by transcription factor networks, central to which are IRF4 and IRF8. These were thought to act redundantly during B cell development in the bone marrow, with their functions diverging in the periphery where IRF4 limits the number of marginal zone B cells and is required for germinal center responses and plasma cell differentiation. Because of IRF4’s unique role in mature B cells, we hypothesized that it may also have functions earlier in B cell development that cannot be compensated for by IRF8. Indeed, we find that IRF4 has a unique role in upregulating the pre-B cell marker CD25, limiting IL-7 responsiveness, and promoting migration to CXCR4 such that IRF4-deficient mice have a partial block at the pre-B cell stage. We also find that IRF4 acts in early transitional B cells to restrict marginal zone B cell development, as deletion of IRF4 in mature B cells with CD21-cre impairs plasma cell differentiation but has no effect on marginal zone B cell numbers. These studies highlight IRF4 as the dominant IRF family member in early B lymphopoiesis.

## Introduction

During B cell development in the bone marrow, a tightly controlled series of events ensures that functional B cells are produced that can recognize an almost infinite number of foreign antigens while limiting the escape of autoreactive B cells into the periphery [reviewed in (1, 2)]. The immunoglobulin (Ig) genes are rearranged sequentially and tested during this process, beginning with the Ig heavy chain at the pro-B cell stage. Successful pairing of the expressed heavy chain with the surrogate light chain promotes a proliferative expansion at the large pre-B cell stage. IL-7 also promotes expansion of pro-B and large pre-B cells. At the small pre-B cell stage, cells exit the cell cycle and rearrange the Ig light chain genes. The transition between the large and small pre-B stage is driven by a particular mode of pre-BCR signaling dependent on the adaptor molecule BLNK (also known as SLP-65) (3, 4), loss of IL-7 signaling (3, 5), and CXCL12/CXCR4 responses (5, 6). After light chain rearrangement, cells transition to the immature B stage where they express the BCR on the surface and are tested for autoreactivity by central tolerance mechanisms.

These developmental transitions are coordinated by a complex network of transcription factors (3). Among these, the related factors IRF4 and IRF8 play a critical role. Previous studies have shown these to be redundantly required for early B cell developmental events. Mice lacking both IRF4 and IRF8 have an almost complete block at the large pre-B stage, while single knockouts have relatively normal numbers of peripheral B cells (7). IRF4/IRF8 double deficient pre-B cells proliferate extensively in response to IL-7, fail to rearrange Ig light chain genes due to impaired germline transcription, and have reduced expression of CXCR4 (5, 6, 8). *In vitro*, reconstitution of IRF4/IRF8 double knockout pre-B cells with either IRF4 or IRF8 allowed the cells to undergo Ig light chain rearrangement upon IL-7 withdrawal (8). This indicates that both IRF4 and IRF8 are both capable of exerting this function and act in a B cell intrinsic manner to do so.

While IRF4 and IRF8 work together in early B lymphopoiesis, IRF4 has a unique role in the periphery in terms of both B cell subset distribution and differentiation. Two major subsets of mature B cells are marginal zone (MZ) B cells, which respond rapidly to T-independent antigens, and follicular B cells, which are activated in the context of T cell help to form germinal centers and produce high affinity, long lived class switched humoral responses. In the absence of IRF4, splenic B cells are present in relatively normal numbers but are skewed towards MZ B cells and away from follicular B cells (9). This is thought to be due to increased Notch2 signaling (9, 10), which is necessary for MZ B cell development and localization (11–13). IRF4 is also required in B cells for germinal center formation and plasma cell differentiation (14–16).

Because of IRF4’s unique role in mature B cells, we hypothesized that it may also have some functions earlier in B cell development that cannot be compensated for by IRF8. This is supported by the observation that loss of several other components of IL-7 and pre-BCR signaling pathways causes defects in pre-B cells and partial blocks in B lymphopoiesis, but still allows the eventual development of relatively normal numbers of mature B cells (17–19). Indeed we find that IRF4 deficient mice have a partial block at the pre-B stage, accompanied by reduced expression of the pre-B cell marker CD25, increased responsiveness to IL-7, and impaired migration towards CXCL12. We also show that IRF4 acts prior to the mature B cell stage to control MZ B cell numbers.

## Methods

### Mice

IRF4fl/fl (14) and CD21-cre (20) mice were obtained from Jackson Labs (catalog numbers 009380, and 006368, respectively) and crossed to each other to generate mice with IRF4 deleted in mature B cells. We also attempted to generate mice lacking IRF4 in all B cells using mb1-cre (Jackson Labs catalog number 020505). However, these studies were unsuccessful because, the mb1-cre caused frequent deletion of IRF4 in the germline as has been described for some loci (21). These germline deleted mice were bred to establish the IRF4-deficient mice. Mice are on the C57BL/6 background. Mice were sex and age matched and littermate controls were used whenever possible. All experiments were approved by the University of Texas Southwestern Medical Center Institutional Animal Care and Use Committee.

### Flow Cytometry

Single cell suspensions of bone marrow and spleen depleted of red blood cells and cell cultures (see below) were and stained extracellularly with combinations of the following antibodies coupled to PE, PerCP, APC, and/or biotin [plus streptavidin APC (Tonbo)]. Bone marrow and IL-7 cultures: B220 (eBioscience), CD43 (BD Pharmingen), IgM (BD Pharmingen), CD25 (Tonbo), and CD93 (eBioscience). Spleen and LPS cultures: B220 (eBioscience), CD21 (BD Pharmingen), CD23 (BioLegend), CD93 (eBioscience), and CD138 (BD Pharmingen). For intracellular staining, cells were first stained extracellularly with combinations of the above antibodies, then stained intracellularly with antibodies against IRF4 (Invitrogen), IRF8 (eBioscience), IgM (BD Pharmingen), VpreB (Biolegend) or isotype control using the FoxP3 Buffer Set (eBioscience). Samples were run on a FACS Calibur (Becton Dickinson) and analyzed with FlowJo software (TreeStar).

### IL-7 Cultures

Following lysis of red blood cells, bone marrow cells were cultured at 2 x 10^6^/ml in complete RPMI (RPMI 1640 + 10% FBS + L-glut + pen/strep + β-ME) and 10ng/ml IL-7 (R&D Systems) for 5 days and then subjected to flow cytometry. In some experiments, cells were then washed, resuspended in complete RPMI, and plated at 10^6^/100ul in the top well of a transwell plate (Corning). 600 ul of c-RPMI with 0.1ug/ml CXCL12 (R&D Systems) was placed in the bottom well. After 3.5 hours at 37°, cells were collected from the bottom well, counted, and subjected to flow cytometry.

### Plasma Cell Differentiation

Splenocytes were subjected to red blood cell lysis and B cells were then purified by depletion of CD43+ cells with magnetic beads according to the manufacturer’s instructions (Miltenyi Biotech). Cells were then cultured at 10^6^/ml in complete RPMI plus 5 ug/ml LPS (Sigma) for 3 days and then subjected to flow cytometry.

### Real Time PCR

Splenocytes were subjected to red blood cell lysis and B cells were then purified by depletion of CD43+ cells with magnetic beads according to the manufacturer’s instructions (Miltenyi Biotech). RNA was prepared using the RNeasy Mini kit (Qiagen) and cDNA subsequently generated using the High Capacity cDNA Reverse Transcription kit (Thermofisher). Quantitative PCR was then performed on a Biorad CFX96 Real-Time System machine (Biorad) using the Taqman reagents for IRF4 and GAPDH (Thermofisher). Data were normalized to GAPDH using the delta Ct method.

### Statistical Analysis

Statistical analysis was performed using Graph Pad Prism. The Student’s t-test or Mann-Whitney test were used for normally and non-normally distributed data, respectively. p < 0.05 is considered significant.

## Results

### Unique Role for IRF4 in Early B Cell Development

**Figure 1:**
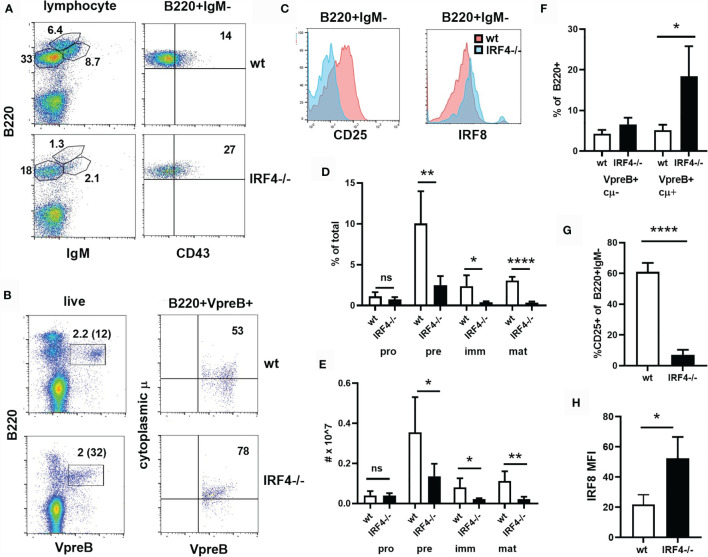
Partial block beyond the preBCR+CD25- stage of B cell development in IRF4-deficient bone marrow. **(A)** Bone marrow was stained with antibodies against B220, IgM and CD43. The gate used is shown above the plots, and the frequency of cells in the relevant gates is indicated. **(B)** Bone marrow was stained extracellularly with anti-B220 and intracellularly with anti-VpreB and anti-IgM heavy chain (cytoplasmic μ or cμ). The gate used is shown above the plots. The frequency of cells in the relevant gates is indicated [on the left plot as a percentage of total and of B220+ (in parentheses)]. **(C)** Bone marrow was stained with antibodies against B220, IgM, and CD25 or intracellular IRF8. CD25 and IRF8 histograms are shown for B220+IgM- cells. Red = wt, blue = IRF4-deficient. **(D, E)** The frequency **(D)** and total number **(E)** of pro-B (B220+IgM-CD43+), pre-B (B220+IgM-CD43-), immature B (B220^med^IgM+), mature B (B220^hi^) cells in the bone marrow of wt (open bars) and IRF4-deficient (filled bars) mice, gated as in **(A)**. Data represent mean +/- SD, n= 5. ns, not significant; *p < 0.05, **p < 0.01, ****p < 0.0001 by Student’s t-test. **(F)** The frequency of VpreB+cμ- and VpreB+cμ+ cells among B220+ cells is indicated, gated as in **(B)**. Data represent mean +/- SD, n = 4. *p < 0.05 by Student’s t-test. **(G)** The frequency of CD25+ cells among B220+IgM- cells is shown. Data represent mean +/- SD, n = 6. ****p < 0.0001 by Student’s t-test. **(H)** IRF8 gMFI in B220+IgM- cells is shown. Data represent mean +/- SD, n = 3. *p < 0.05 by Student’s t-test.

To determine whether IRF4 has unique roles early in B cell development, we analyzed bone marrow B cells from IRF4-deficient mice by flow cytometry. While B220+CD43+IgM- pro-B cells were present in normal numbers, there was a significant reduction in the numbers of B220+CD43-IgM- pre B cells, B220+CD43-IgM+ immature B cells, and B220hiIgM+ mature B cells in IRF4-deficient mice (**Figures 1A, D, E**). The frequency of B lineage cells expressing the pre-BCR (both VpreB and cytoplasmic μ) was increased in IRF4 deficient mice (**Figures 1B, F**), while there were fewer CD25+ cells among B220+IgM- cells (**Figures 1C, G**). These data indicate a partial block beyond the pre-BCR+CD25- stage of development. IRF8 was expressed at twice the normal level in B220+IgM- cells from IRF4-deficient mice (**Figures 1C, H**). IRF8 is thus unable to compensate fully for IRF4 in early B cell development.

**Figure 2:**
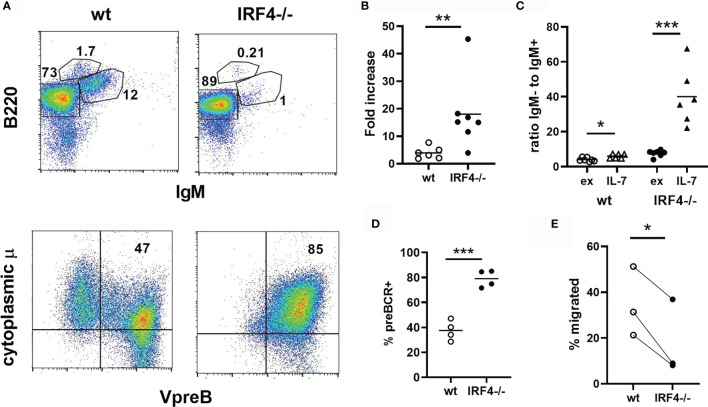
Increased expansion and impaired differentiation of pre-B cells in IRF4-deficient IL-7 cultures. Bone marrow from wild type (open symbols) or IRF4-deficient (filled symbols) BM was cultured in 10 ng/ml IL-7 for 5 days. **(A)** Cells were subsequently stained either extracellularly with antibodies against B220 and IgM (top, lymphocyte gate), or extracellularly with anti-B220 and intracellularly with anti-VpreB and anti-IgM heavy chain (bottom, B220+ gate). **(B)** The fold increase in the total number of B220+IgM- cells in IL-7 cultures relative to the input. Each symbol represents an individual mouse, the bar the mean. **p < 0.01 by Mann-Whitney test. **(C)** The ratio of B220+IgM- to immature B cells in *ex vivo* bone marrow (ex) and day 5 IL-7 cultures (IL-7) is indicated. Each symbol represents an individual mouse, the bar the mean. *p < 0.05, ***p < 0.001 by Student’s t-test. **(D)** The frequency of pre-BCR expressing (VpreB+cytoplasmic μ+) cells among B220+ cells is indicated, gated as in **(A)**. Each symbol represents an individual mouse, the bar the mean. ***p < 0.001 by Student's t-test. **(E)** Cells from day 5 IL-7 cultures were plated in a transwell insert with 0.1ug/ml CXC12 in the bottom chamber. The percentage of B220+IgM- cells that migrated after 3.5 hrs is indicated. Each symbol represents an individual mouse, and each line connects results from an individual experiment. *p < 0.05 by paired t-test.

Stimulation of bone marrow cells with IL-7 results in the proliferative expansion of pro- and pre-B cells. This response was enhanced in the absence of IRF4 (**Figures 2A, B**). In wild type IL-7 cultures, the ratio of B220+IgM- cells to immature B cells was only mildly elevated (1.8 fold) compared to ex vivo bone marrow, indicating that some of the expanding pro- and pre-B cells differentiate into immature B cells in the cultures. In contrast, very few IgM+ cells were present in IL-7 cultures of IRF4-deficient bone marrow, and the ratio of B220+IgM- cells to immature B cells was increased dramatically (6.7 fold) upon IL-7 culture (**Figures 2A, C**). The vast majority of B lineage cells in the IRF4-deficient IL-7 cultures expressed the preBCR (**Figures 2** **A, D**). The ability to respond to the chemokine CXCL12 contributes to the differentiation of immature B cells both by promoting migration away from IL-7 rich niches and by sending a direct signal (5, 6). Responses to CXCL12 are impaired in the combined absence of IRF4 and IRF8 (5, 6). We observed that B220+IgM- B cells from IRF4-deficient IL-7 cultures demonstrated reduced migration towards CXCL12 than their wild type counterparts (**Figure 2E**). Thus, IRF4 deficiency alone results in enhanced expansion of pre-BCR+ B cells in the presence of IL-7 and reduced differentiation of these cells into immature B cells.

### IRF4 Acts Prior to the Mature B Cell Stage to Control Marginal Zone B Cell Numbers

**Figure 3:**
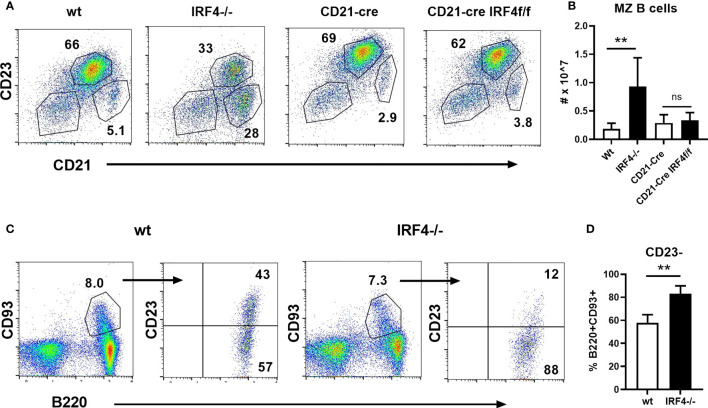
Normal MZ B cell numbers in CD21-cre.IRF4f/f mice. **(A, B)** Splenocytes from wild type, IRF4-deficient, CD21-cre, and CD21-cre.IRF4f/f mice were stained with antibodies against B220, CD21, and CD23. **(A)** The frequency of follicular (CD21+CD23+) and MZ (CD21hiCD23lo/-) in the B220+ gate is shown. **(B)** The total number of MZ B cells gated as in **(A)** is shown. Data represent mean +/- SD, n = 3-6. **p < 0.01, ns, not significant by Student’s t-test. **(C, D)** Splenocytes from wild type and IRF4-deficient mice were stained with antibodies against B220, CD93, and CD23. Representative flow cytometry plots are shown in **(C)**, while **(D)** indicates the frequency of B220+CD93+ transitional cells that lack CD23. Data represent mean +/- SD, n = 6. **p < 0.01 by Student’s t-test.

Despite the observed defects in early B cell development described above, peripheral B cells are able to develop in IRF4-deficient mice. Of particular interest, IRF4 has a unique role in limiting the number of MZ B cells in the spleen. Consistent with previous reports (9, 14, 15), we find that MZ B cells (CD21hiCD23lo/-) are increased both in frequency and number in the absence of IRF4 (**Figures 3** **A, B**). To determine whether this effect is independent of IRF4’s role earlier in B cell development, we generated CD21-cre.IRF4fl/fl mice to delete IRF4 in mature B cells (20). Bone marrow B cell development was normal as expected (**Supplemental Figure 1**). Surprisingly, there was no difference in either the frequency or numbers of MZ B cells in CD21-cre IRF4 fl/fl mice (**Figures 3A, B**). Another important function of IRF4 in mature B cells, plasma cell differentiation (14), was impaired in these mice, and IRF4 mRNA levels were significantly reduced (**Supplemental Figure 1**). At the protein level, IRF4 was decreased similarly in CD21-cre.IRF4fl/fl and IRF4-deficient MZ B cells. IRF4 protein expression was lower in wild type follicular B cells as previously shown (16, 22), and was further reduced in CD21-cre.IRF4fl/fl and IRF4-deficient cells (**Supplemental Figure 1**). IRF8 was slightly elevated in both B cell subsets in the absence of IRF4 (**Supplemental Figure 1**). These results suggest that IRF4 acts prior to the expression of CD21, rather than within MZ B cells themselves, to control MZ B cell numbers. It has recently been reported that a subpopulation of T1 B cells is committed to the MZ fate based on adoptive transfer studies (10). In support of a role of IRF4 in T1 cells, the frequency of T1 cells (B220+CD93+CD23-) among splenic transitional cells (B220+CD93+) is elevated in IRF4-deficient mice (**Figures 3C, D**).

## Discussion

Based on the study of IRF4/IRF8 double knockout mice and the reconstitution of pre-B cells from these animals with either IRF4 or IRF8, it has been thought that IRF4 and IRF8 are redundant for early B cell developmental events. Here, by characterizing IRF4-deficient mice, we find that IRF8 does not compensate, or can only partially compensate, for IRF4. Expression of CD25 in B220+IgM- cells was almost completely dependent on IRF4 alone. IRF4 also has a dominant role in suppressing proliferation in response to IL-7, as demonstrated by the increased expansion of B220+IgM- cells in IL-7 cultures of IRF4-deficient bone marrow. Finally, we observed a reduction in IgM+ immature B cells both ex vivo and in IL-7 cultures of IRF4-deficient mice. However, IgM+ immature B cells were still present in these mice, and splenic B cell numbers are not decreased although they are skewed towards MZ B cells and away from follicular B cells. In contrast, IRF4-/-IRF8-/- mice lack all immature and mature B cells in both the bone marrow and the spleen due to a failure to undergo Ig light chain recombination. Thus, while IRF4 contributes to light chain assembly and subsequent IgM expression, IRF8 can compensate for this activity to allow for the development of peripheral B cells in the absence of IRF4.

One caveat to the interpretation of these results is that the mice used for the bone marrow studies here were whole body knockouts. We attempted to generate mice lacking IRF4 throughout the B lineage using mb1-cre. However, these studies were unsuccessful because the mb1-cre caused frequent deletion of IRF4 in the germline as has been described for some loci (21). *In vitro* transduction of purified IRF4-/-IRF8-/- pre B cells with IRF4 results in upregulation of CD25 and, upon IL-7 withdrawal, light chain rearrangement and surface IgM expression (5, 8). This, as well as the links to B cell intrinsic signaling pathways discussed below, suggest that IRF4 has a B cell intrinsic role in the progression of B lineage cells beyond the pre-BCR+ CD25-stage. However, we cannot rule out a role for non-B cell expressed IRF4 in regulating proliferation or B lineage cell numbers.

Several of the early B cell developmental defects in IRF4-deficient mice resemble those of mice lacking either of the pre-BCR signaling components Btk or BLNK (also known as SLP-65). B220+IgM- bone marrow cells from both Btk-/- and BLNK-/- mice have reduced CD25 expression, increased pre-BCR expression, and increased expansion in response to IL-7 (17–19). Similarly, Btk and BLNK knockout animals have reduced light chain recombination but are able to develop IgM+ peripheral B cells (17–19). This supports a model in which a pre-BCR/Btk/BLNK signaling module preferentially signals *via* IRF4, rather than IRF8, to control B cell development in the bone marrow. Indeed, BLNK has been shown to mediate pre-BCR induced IRF4 expression (3, 23). This is due to its attenuation of PI3K/Akt signaling and subsequent upregulation of Foxo1, which in turn drives IRF4 expression (3, 23). Intriguingly, inhibiting PI3K signaling increases IRF4, but not IRF8 expression, in BLNK-deficient pre-B cells (23). In a feed-forward loop, IRF4 but not IRF8 can promote Pax5 expression (23) which, together with Foxo1, upregulates BLNK levels (3, 24). The participation of IRF4 but not IRF8 in these amplification mechanisms may account for the more dominant role of IRF4 in early B cell development.

In the periphery, IRF4-deficient mice have an increase in MZ B cells. This is thought to be a result of increased Notch signaling which is known to drive the MZ B cell fate decision and promote retention in the marginal zone (9). We found that CD21-cre.IRF4f/f mice had normal numbers of MZ B cells. This suggests that IRF4 acts prior to the upregulation of CD21 to drive MZ B cell expansion. It has recently been shown that T1 B cells expressing cell surface Adam10, a metalloproteinase required for the cleavage and activation of Notch2 (13), give rise to MZ B cells upon adoptive transfer (10). This study also reported that IRF4-deficient mice have a greater frequency of T1 cells expressing surface Adam10 compared to their wild type counterparts (10). Here, we find that IRF4-deficient transitional B cells are enriched in T1 cells. Taken together, these data support a model in which loss of IRF4 prior to the mature B stage results in a skewing of transitional B cells towards a MZ B-committed T1 subset. MZ B cells are also increased when Foxo1 is deleted with CD19-cre but not CD21-cre (25). As they do in pre-B cells (3, 23), Foxo1 and IRF4 may act together to limit T1 cell commitment to the MZ B fate. Alternatively, and not mutually exclusively, a low level of residual IRF4 in CD21-cre.IRF4f/f B cells may be sufficient to limit MZ B cell expansion but not to drive plasma cell differentiation, as dosage of IRF4 has been shown to affect both its binding partners and its downstream function (15).

In summary, we have identified a unique role for IRF4 during early B lymphopoeisis that cannot be compensated for by IRF8. We also find that IRF4 acts prior to the expression of CD21 to limit MZ B cell development. Future studies comparing IRF4 and IRF8 targets in pre-B cells and identifying novel IRF4 targets in T1 cells will provide further clarity on the role of this critical transcription factor in B cell development.

## Data Availability Statement

The original contributions presented in the study are included in the article/**Supplementary Material**. Further inquiries can be directed to the corresponding author.

## Ethics Statement

The animal study was reviewed and approved by UT Southwestern Institutional Animal Care and Use Committee (IACUC).

## Author Contributions

KO performed experiments, analysed data, and edited the manuscript. AS designed experiments, analysed data, and wrote the manuscript. All authors contributed to the article and approved the submitted version.

## Funding

This work was supported by NIH grant AI137746 to AS. AS is a Southwestern Medical Foundation Scholar in Biomedical Research and holds the Peggy Chavellier Professorship in Arthritis Research and Treatment.

## Conflict of Interest

The authors declare that the research was conducted in the absence of any commercial or financial relationships that could be construed as a potential conflict of interest.

## Publisher’s Note

All claims expressed in this article are solely those of the authors and do not necessarily represent those of their affiliated organizations, or those of the publisher, the editors and the reviewers. Any product that may be evaluated in this article, or claim that may be made by its manufacturer, is not guaranteed or endorsed by the publisher.
