# Supplementary material for: IRF4 Has a Unique Role in Early B Cell Development and Acts Prior to CD21 Expression to Control Marginal Zone B Cell Numbers
Source: Front Immunol. 2021 Nov 22;12:779085. doi: 10.3389/fimmu.2021.779085 (PMC8645892; doi:10.3389/fimmu.2021.779085)
Supplement: Supplementary file 1 [file DataSheet_1.pdf]

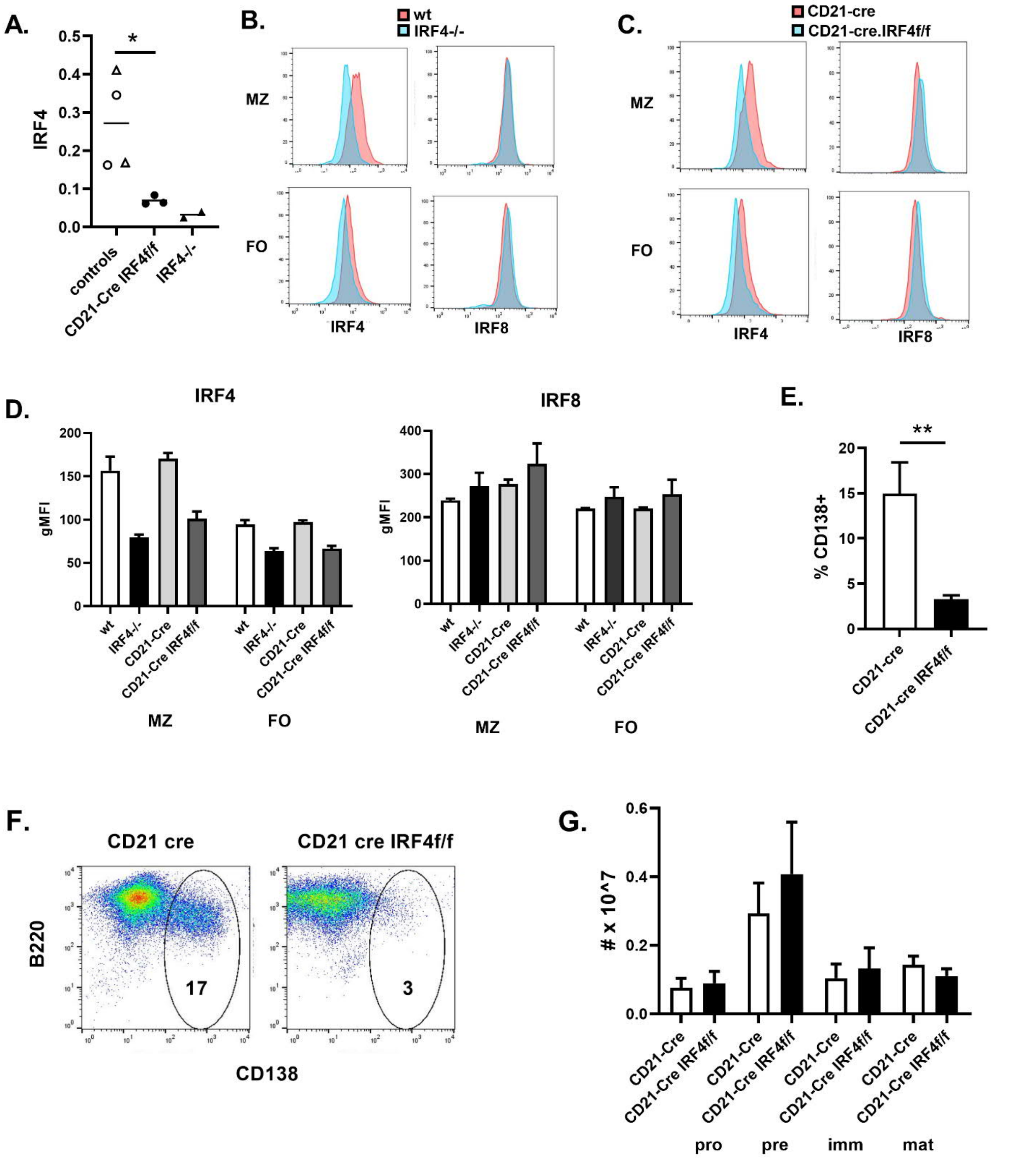

Supplemental Figure 1

A) Real time quantitative PCR of IRF4 levels in purified splenic B cells from mice of the indicated genotypes. Results are normalized to GAPDH. Controls include wild type (open triangles) and CD21-cre (open circles) cells. Each symbol represents an individual mouse. *p < 0.05 by Student's t=test. B, C) Splenocytes were stained extracellularly with antibodies against CD21 and CD23 and intracellularly with anti-IRF4 or IRF8. IRF4 and IRF8 levels are shown for

MZ (CD21hi CD23lo/-) and follicular (CD21+CD23+) B cells. Wild type (red) vs. IRF4-deficient (turquoise) mice are shown in (B), and CD21-cre (red) vs CD21-cre.IRF4f/f (turquoise) mice in (C). D) The gMFI of IRF4 and IRF8 is shown for MZ and FO B cells of the indicated genotypes. Data are shown as mean +/- SD, n = 2. E, F) Purified splenic B cells from mice of the indicated genotypes were stimulated with 5 ug/ml LPS for 72 hours and stained with antibodies against B220 and CD138. The frequency of CD138+ plasmablasts is indicated in a representative flow cytometry plot (F) and as mean +/- SD for n = 2 CD21-cre and 3 CD21-cre.IRF4f/f (E). **$p < 0.01$ by Student's t-test. G) The total number of pro, pre, immature, and mature B cells (gated as in Figure 1) in the bone marrow of CD21-cre and CD21-cre.IRF4f/f mice is shown as mean +/- SD, n = 2.
